# Facile synthesis of P(EDOT/Ani) : PSS with enhanced heat shielding efficiency *via* two-stage shot growth†

**DOI:** 10.1039/c8ra01122b

**Published:** 2018-04-09

**Authors:** Chanil Park, Soeun Im, Wonseok Cho, Yunryeol Kim, Jung Hyun Kim

**Affiliations:** Department of Chemical and Biomolecular Engineering, Yonsei University Yeonsero 50, Seodaemun-gu Seoul 120-749 Republic of Korea jayhkim@yonsei.ac.kr

## Abstract

Poly(3,4-ethylenedioxythiophene/aniline) : poly(styrene sulfonate), P(EDOT/Ani) : PSS, with enhanced absorption of near infrared light, was prepared by oxidative polymerization. We demonstrated that a two-stage shot growth process optimizes the absorption of the polymer in the near infrared region *via* a controlled monomer addition time. In other words, the optical properties of the polymer complex were improved by controlling the time intervals of aniline monomer addition. P(EDOT/Ani) : PSS was characterized by Fourier transform infrared spectroscopy (FT-IR) and X-ray photoelectron spectroscopy (XPS). The shielding efficiency of the P(EDOT/Ani) : PSS films was calculated by using data from ultraviolet, visible, and near infrared (UV-vis-NIR) spectroscopy. The introduction of polyaniline to PEDOT increased the absorption in the near infrared area. In comparison with PEDOT : PSS film, the total shielding efficiency of the P(EDOT/Ani) : PSS film increased to 65.8% from 54.6% at 60% transmittance. The maximum NIR shielding efficiency (SE_NIR_) of the film is 92.7% and the transmittance is 46.5%. Also, large-scale P(EDOT/Ani) : PSS film was fabricated using roll-to-roll slot-die equipment and a heat shielding test of the film was conducted by measuring the temperature variation, in order to prove the enhanced heat shielding effect. P(EDOT/Ani) : PSS prepared by a two-stage shot growth system showed excellent potential as a heat shielding material.

## Abstract 2

Poly(3,4-ethylenedioxythiophene/aniline) : poly(styrene sulfonate), P(EDOT/Ani) : PSS, with enhanced absorption of near infrared light, was prepared by oxidative polymerization.

## Graphical abstract

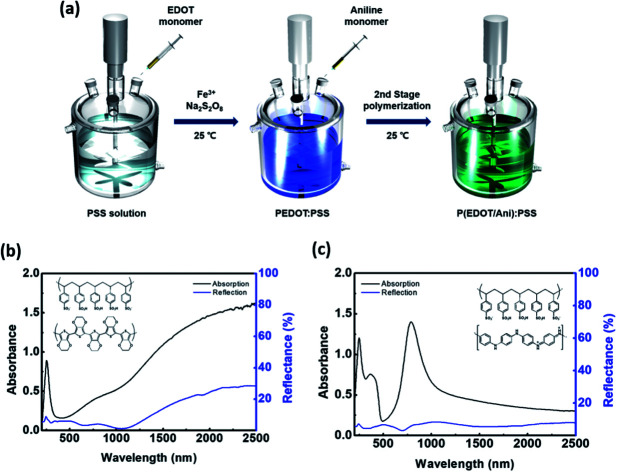

## Introduction

Conjugated polymers have attracted intense interest for applications in photonics, sensing, light emitting diodes and energy harvesting.^1–5^ In particular, poly(3,4-ethylenedioxythiophene) (PEDOT) has been actively studied due to its excellent electrical and optical properties and its environmental stability. PEDOT has very poor solubility in water, but when polystyrene sulfonate (PSS), a linear ionic polymer, is introduced, its solubility is improved. PSS also acts as a dopant and enhances the electrical conductivity of PEDOT. Therefore PEDOT : PSS has been used in various applications such as a hole transport layer for solar cells, thermoelectric devices, and transparent electrodes.^6–9^ In addition, PEDOT : PSS can be used as a heat shielding film material because of its high optical absorption in the near infrared (NIR) region. A low band gap for the conjugated polymers contributes to excellent absorption at longer wavelengths of light.^10^

Recently, energy conservation has been an important issue and research has been conducted in many related fields. Ordinary glass typically has high transmittance and low reflectance in the visible and near infrared regions. To prevent energy losses inside buildings and vehicles, it is necessary to block sunlight in the summer and to prevent loss of indoor heat in the winter. Therefore, many studies have been carried out to develop smart window coating materials with high absorption in the infrared region. Inorganic heat shielding materials such as interstitially doped tungsten oxide (M_*x*_WO_3_, M = Li, Na, K, Rb, or Cs) or indium tin oxide (ITO) are the main types of materials that have been investigated.^11^ Wang *et al.* fabricated NIR shielding films using F–TiO_2_ and K_*x*_WO_3_. The K_*x*_WO_3_ was in the form of nanorods as the NIR shielding materials were synthesized with Na_2_WO_4_.^12^ Zhang *et al.* prepared transparent heat insulation coatings using antimony doped tin oxide (ATO) dispersions.^13^ These inorganic coating materials are promising alternatives for low-emissivity (low-E) glass for energy efficient windows. But they have the disadvantages of having a high cost and a difficult coating process due to the requirement for a high-temperature treatment process and many additives for coating stability. A heat shielding film coated with organic materials is more cost-effective and simpler to manufacture. Chen *et al.* synthesized polypyrrole (PPy) nanoparticles and polyacrylic acid (PAA) resin for UV/NIR shielding film.^14^ In this study, we investigated the enhanced heat shielding effect of PEDOT and polyaniline (PAni) composites, synthesized by oxidative polymerization.^15^ By introducing PAni to PEDOT : PSS, absorption in the NIR region significantly improved with a minimal loss in transmittance. In addition, the P(EDOT/Ani) : PSS film have an excellent coating ability without the use of additives such as leveling agents and binders, and the film is stable under high temperature (85 °C) or high temperature/humidity (85 °C/85% R.H.) conditions. Finally, we fabricated a large area P(EDOT/Ani) : PSS film (500 mm in width × 150 m in length) with improved heat shielding efficiency using roll-to-roll slot-die equipment.

## Experimental

### Materials

3,4-Ethylenedioxythiophene (EDOT, 97%), aniline (Ani, ≥99.5%), sodium persulfate (Na_2_S_2_O_8_, ≥99.0%), iron(iii) sulfate [Fe_2_(SO_4_)_3_, 97%], poly(sodium 4-styrenesulfonate) (PSS), and isopropyl alcohol (IPA), dimethyl sulfoxide (DMSO), cation exchange resin, and anion exchange resin were purchased from Sigma Aldrich Co., Yongin-Si, Gyeonggi-do, Korea. All reagents were used as received without further purification. Distilled deionized (DDI) water was used throughout the experiments.

### Preparation of poly(3,4-ethylenedioxythiophene/aniline) : polystyrene sulfonate [P(EDOT/Ani) : PSS] and poly(aniline/3,4-ethylenedioxythiophene) : polystyrene sulfonate [P(Ani/EDOT) : PSS]

An aqueous dispersion of poly(sodium-4-styrenesulfonate) (PSS, 85 mL) was bubbled with inert nitrogen gas (N_2_, 99.999%) for 60 min. 3,4-Ethylenedioxythiophene (EDOT) monomer (1.5 mmol) was added dropwise to the PSS solution with stirring. After stirring the reaction mixture for 10 min, the oxidizing reagents, sodium persulfate (0.4 mmol) and iron(iii) sulfate (0.017 mmol), were added at room temperature for 23 h. At various time intervals, aniline (Ani) (1.5 mmol) was added dropwise to the mixture as the second monomer. To remove residual ions, the product was mixed with cation and anion exchange resins at room temperature for 1 h. Finally, the mixture was filtered and a dark green liquid solution was obtained, consisting of PEDOT : PSS and PAni : PSS.

P(Ani/EDOT) : PSS was also synthesized to compare the reaction kinetic of the polymer complex. P(Ani/EDOT) : PSS was synthesized under the same conditions except for the addition sequence of monomers.

### Characterization

The FT-IR spectrum of P(EDOT/Ani) : PSS solution was measured by a FT-IR spectrometer in ATR mode (Bruker, model Vertex 70). The UV-vis-NIR absorbance of P(EDOT/Ani) : PSS films was measured by a spectrophotometer (JASCO Corporation, model V-770). X-ray photoelectron spectroscopy was performed using an X-ray photoelectron spectrometer (K-alpha, Thermo VG) equipped with a 180° spherical sector analyzer and monochromated Al X-ray source (Al Kα line: 1486.6 eV). Infrared bulbs (250 W, PHILIPS) and thermometers were used to obtain a temperature variation curve for the heat shielding films.

## Results and discussion

### Synthesis of P(EDOT/Ani) : PSS *via* two-stage shot growth

**Fig. 1:**
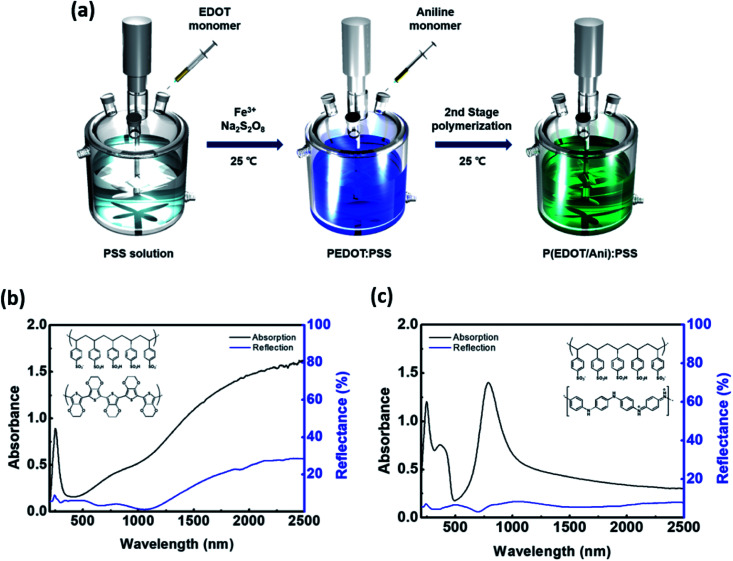
(a) Schematic for synthesis of P(EDOT/Ani) : PSS; (b) UV-vis-NIR spectra of PEDOT : PSS and (c) UV-vis-NIR spectra of PAni : PSS.

P(EDOT/Ani) : PSS was synthesized by oxidative polymerization of EDOT and aniline using sodium persulfate (NaS_2_O_8_) in an aqueous medium. In this work, we introduce a two-stage shot growth process, which is a two-step monomer addition system, for the preparation of a conjugated polymer with enhanced absorbance in the near infrared region. First, the EDOT monomer was polymerized, then aniline was added as a second monomer at certain time intervals. After initiating the EDOT polymer, the aniline addition time was varied in order to achieve a polymer with improved NIR absorbance. Fig. 1 shows the synthesis process for P(EDOT/Ani) : PSS along with the absorbance spectra in the 200–2500 nm range for PEDOT : PSS and PAni : PSS. Typically, PSS displays a strong absorption peak at 230 nm due to π–π* transition of the benzene rings in the PSS system.^16^ The absorption spectrum of PEDOT shows a broad band in the visible and near infrared regions. The characteristic absorbance is associated with polaron or bipolaron states of PEDOT.^17^ In addition, the reflectance of PEDOT gradually increases to 30% in the near-infrared region (1100–2500 nm), as shown in Fig. 1(b). However, a relatively weak absorption of PEDOT at 700–1000 nm wavelength is to be improved for efficient heat shielding effect. A hybridization with PEDOT and PAni is appropriate for increasing absorption in the NIR area. Because, PAni shows strong absorption in the visible region and 780–900 nm wavelength although has a weak absorption at longer wavelengths (see Fig. 1(c) and S1†).^18^ To minimized the absorption offset at wavelengths longer than 1000 nm of PEDOT by PAni, we also optimised polymerization system of P(EDOT/Ani) : PSS using the two-stage shot growth.

**Fig. 2:**
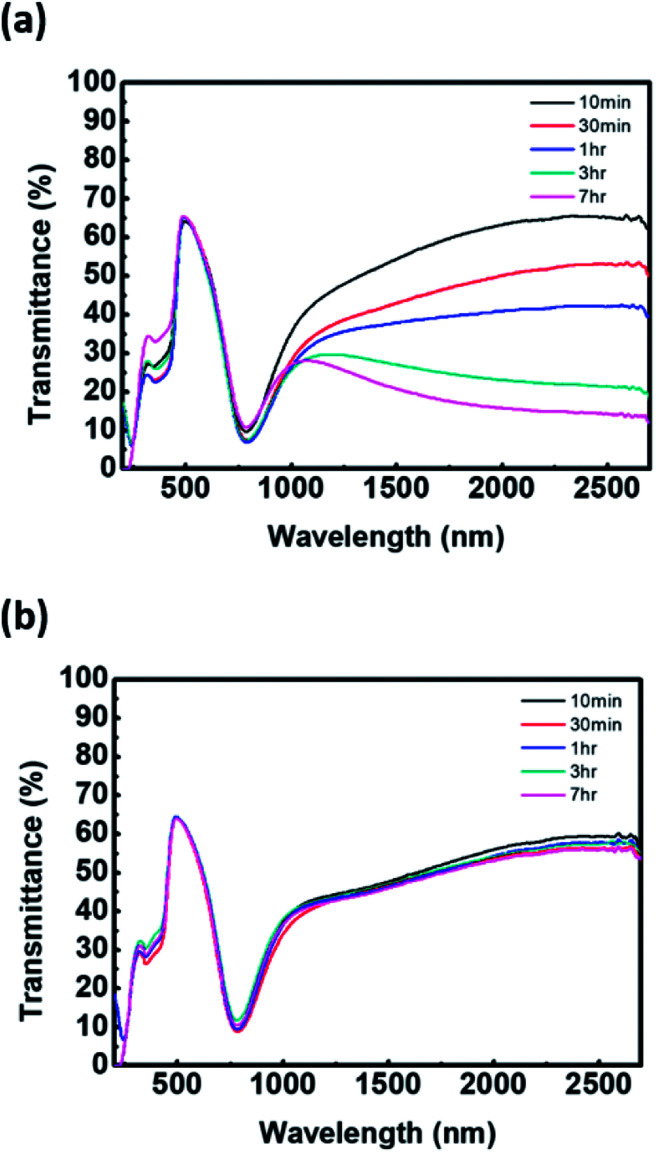
UV-vis-NIR spectrum of (a) P(EDOT/Ani) : PSS films and (b) P(Ani/EDOT) : PSS films for various addition time of second monomer.

**Fig. 3:**
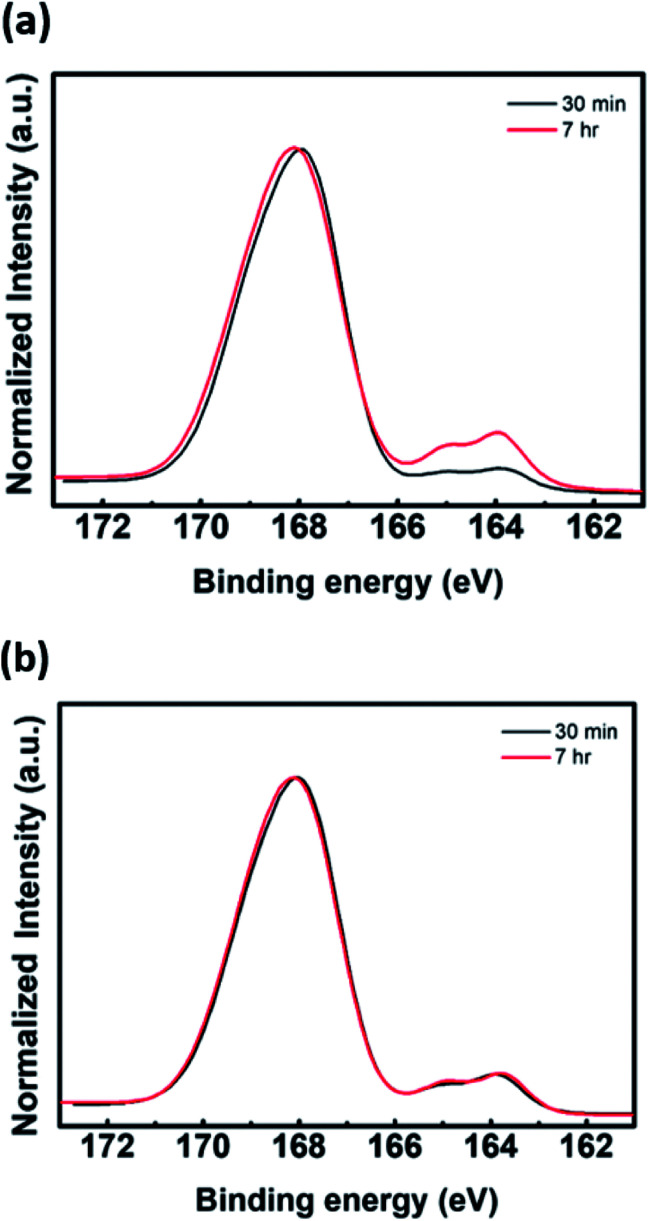
XPS data of (a) P(EDOT/Ani) : PSS films and (b) P(Ani/EDOT) : PSS films.

In terms of the reaction kinetics, the rate constant for the chain propagation of PAni is greater than that for PEDOT. The rate constants of PAni are first order, and the formation of the polymer results in expedited oxidative polymerization.^19^ The reaction rate of PEDOT is sensitive to the reactant concentration because the rate constant is third order. In other words, the oxidative reaction rate of EDOT is very slow.^20^ Hence, addition of aniline as the second monomer can inhibit the chain propagation of PEDOT. As shown in Fig. S2,† FT-IR spectra of P(EDOT/Ani) : PSS with several time intervals of aniline monomer addition reveal a difference in chain growth of PEDOT and PAni. There is no characteristic absorbance peak of polymer in the 1300–4000 cm^−1^ region because the water baseline peak is removed (see Fig. S2 in the ESI†). In contrast, the bands at 602–1030 cm^−1^, assigned to C–O–C deformation and asymmetric and symmetric C–S–C deformation of PEDOT, show that the absorbance increases with increasing interval times of aniline addition.^21,22^ Also, interference with PEDOT growth is clearly confirmed by the optical properties of P(EDOT/Ani) : PSS films. We obtained transmittance spectra of P(EDOT/Ani) : PSS films for different addition times of aniline monomer by using UV-vis-NIR spectroscope. We observed that the transmittance of the films at 1000–2700 nm, a characteristic band for PEDOT, decreased with increasing growth of PEDOT chains. As the addition time of the aniline monomer was delayed, the polymerization of PEDOT proceeded well (see Fig. 2(a)). When the polymerization of aniline was initiated and then EDOT was added to the solution as the second monomer, the growth of PEDOT chains was interrupted (see Fig. 2(b)). Evidence of this interruption was also observed by X-ray photoelectron spectroscopic analysis. A quantitative analysis of the XPS data indirectly demonstrates a difference in the rate of polymerization of EDOT and aniline in heterogeneous systems. The XPS S2p (sulfur) data of P(EDOT/Ani) : PSS and P(Ani/EDOT) : PSS with different addition times for the second monomer are shown in Fig. 3. Typically, the S2p peaks of PEDOT : PSS are observed at about 163–169 eV. The S2p peaks at 163.9 and 165.0 eV come from the sulfur content in the PEDOT. The higher binding energy at 168.0 eV comes from ionic sulfur in the PSS.^9,23^ In the case of P(EDOT/Ani) : PSS, the binding energy at 163.0 and 165.0 eV was greater when the second monomer addition time was 7 h rather than 30 min. In contrast, the S2p peaks of P(Ani/EDOT) : PSS were not changed, because the growth of PEDOT chains was disturbed.

### Heat shielding efficiency of P(EDOT/Ani) : PSS films

In general, conducting polymers absorb visible light well because they have a low band gap. The absorption spectrum of PEDOT is related to the PEDOT oxidation state (polaron or bipolaron state). PEDOT is capable of absorbing light with longer wavelengths than those of visible light. Moreover, the introduction of polyaniline into PEDOT results in an excellent shielding effect in the NIR region. The heat shielding efficiency (SE_heat_) of the P(EDOT/Ani) : PSS films is defined by the following equation:SE_heat_ = 100 − TE

**Fig. 4:**
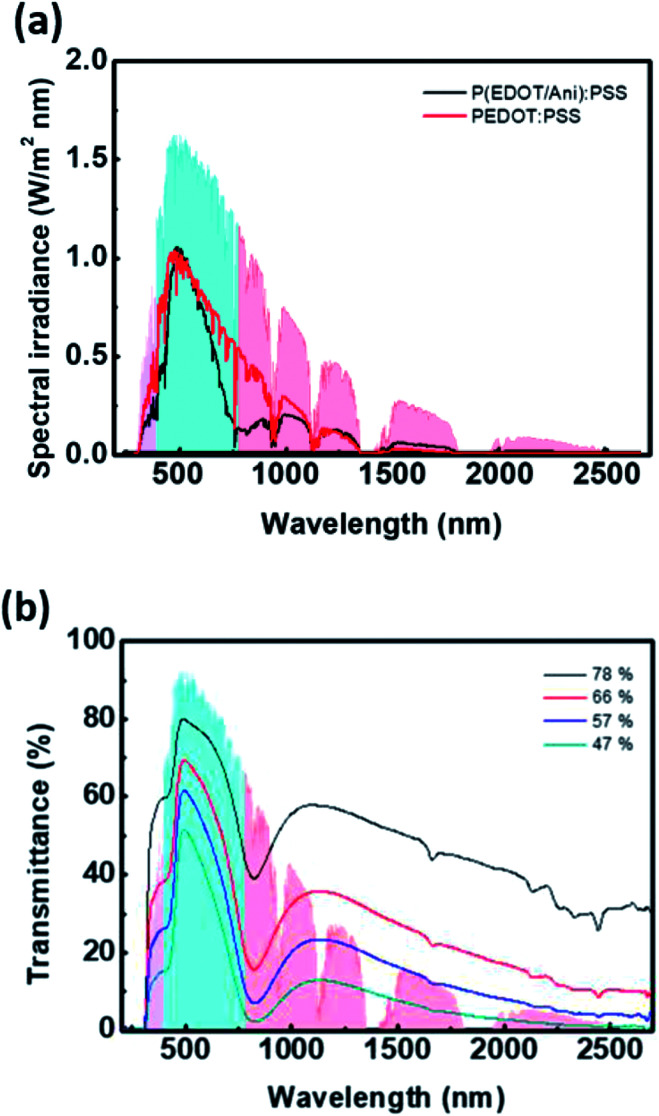
(a) Solar spectral irradiance and transmitted solar energy spectrum under P(EDOT/Ani) : PSS film (black line), pristine PEDOT : PSS film (red line); (b) transmittance spectra of P(EDOT/Ani) : PSS films for various thicknesses of film.

The transmitted efficiency (TE) is obtained by the following equation:
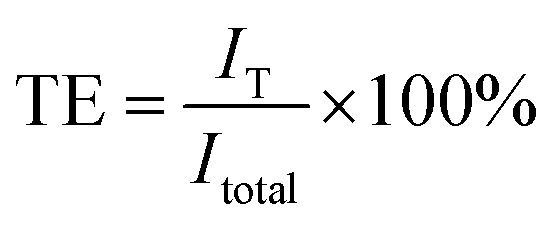


The transmitted intensity (*I*_T_) corresponds to the sum of spectral irradiance for each wavelength:
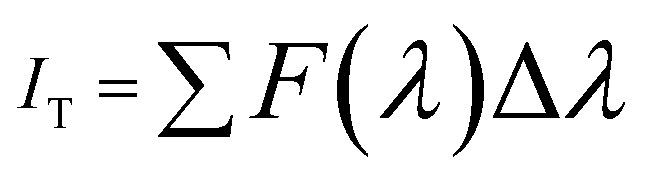


Fig. 4(a) shows the solar spectral irradiance and the transmitted solar energy spectrum of the P(EDOT/Ani) : PSS film. The solar power density was calculated by integrating the spectral irradiance at each wavelength, and was plotted for each region. The ultraviolet (UV) region corresponds to a wavelength from 280 to 400 nm, visible light is 400–780 nm, and near infrared light is 780–2700 nm. As shown in Fig. 4(a), we can predict a reduction of solar energy by P(EDOT/Ani) : PSS film. Also, the shielding effect of NIR light is excellent, but the transmittance in the visible light region is low. These properties result in an enhanced heat shielding effect. The heat shielding efficiency of the 60% transmittance P(EDOT/Ani) : PSS film is 65.8%, which is more than 11.2% higher than the 60% transmittance pristine PEDOT : PSS film. The heat shielding effect of P(EDOT/Ani) : PSS film is dependent on film thickness. Fig. 4(b) shows the transmittance spectra of films at various thicknesses. The transmittance at 550 nm decreased from 77.7% to 46.5% as the thickness increased, but the heat shielding effect increased at the same time. The shielding efficiency in the UV, NIR regions was significantly improved. This result is equivalent to an increase in absorbance as the concentration of the film increases. The calculated shielding efficiency for the P(EDOT/Ani) : PSS films with different thickness is summarized in Table 1. For the 47% transmittance film, SE_UV_, SE_NIR_, and SE_total_ were calculated to be 86.9%, 92.7%, and 79.0%, respectively.

**Table 1:** Shielding efficiency of the P(EDOT/Ani) : PSS films in the UV, visible, and NIR regions

Type	SE at UV^b^ (%)	Transmittance (%) at 550 nm	SE at visible^c^ (%)	SE at NIR^d^ (%)	Total SE (%)
Pristine P^a^	41.2	60.3	42.9	70.6	54.6
T-78	46.1	77.7	29.9	51.1	39.7
T-66	65.9	65.8	46.1	74.1	59.0
T-60	66.1	60.1	54.9	79.1	65.8
T-57	77.1	57.3	56.2	84.9	69.4
T-47	86.9	46.5	67.1	92.7	79.0

a Pristine PEDOT : PSS film.

b 280–400 nm wavelength.

c 400–780 nm wavelength.

d 780–2700 nm wavelength.

### Temperature profile and stability of P(EDOT/Ani) : PSS film

**Fig. 5:**
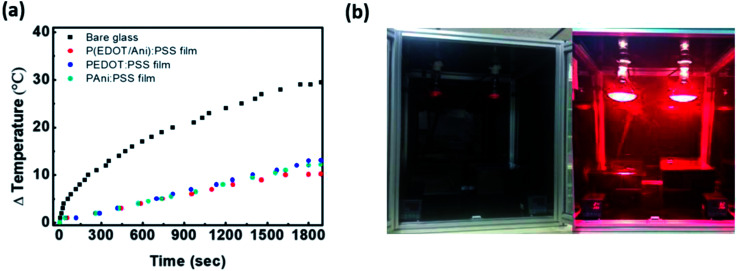
(a) Temperature variation under IR lamp for 30 min: glass alone *vs.* P(EDOT/Ani) : PSS film *vs.* PEDOT : PSS film, PAni : PSS film attached on glass; (b) images of equipment for measurement of the temperature change.

**Fig. 6:**
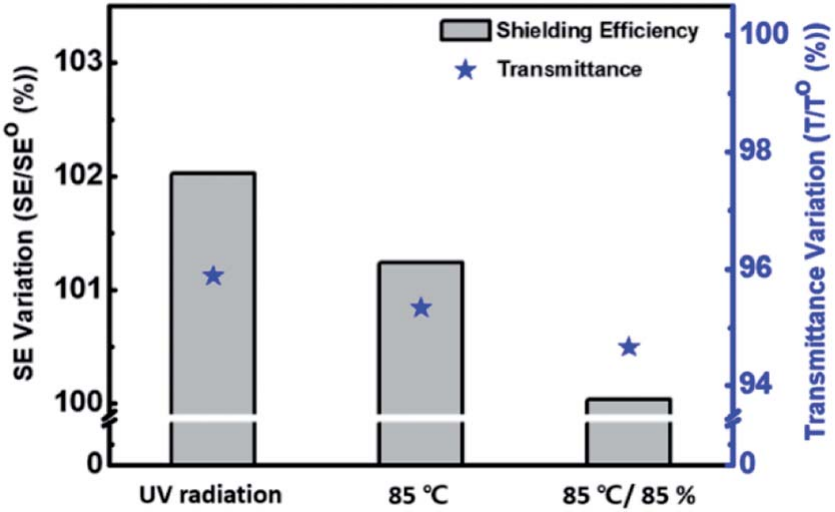
Optical property variation of P(EDOT/Ani) : PSS films after durability test: UV resistance test (254 nm UV radiation for 250 h), high-temperature test (85 °C) for 250 h, high-temperature and humidity test (85 °C/85%) for 250 h.

**Fig. 7:**
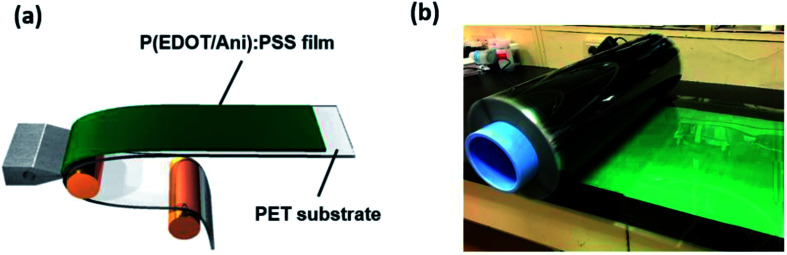
(a) Schematic for fabrication of a large-scale P(EDOT/Ani) : PSS film as heat shielding film; (b) image of the larger-scale P(EDOT/Ani) : PSS film.

To confirm the degree of heat shielding by P(EDOT/Ani) : PSS film, a simple test was conducted using black boxes and infrared lamps (details in the Experimental section). A box measuring 300 mm × 210 mm × 150 mm made of black acrylate was prepared. A thermometer measuring the temperature variation was placed in the black box and the P(EDOT/Ani) : PSS film was attached to a glass window on the open side of the box. The distance between the black box and the infrared lamp was 500 mm. Fig. 5(b) shows the equipment for measurement of the temperature variation with either glass alone or with P(EDOT/Ani) : PSS film attached. After turning on the IR lamps for 1800 s, the temperature with glass alone increased from 22 °C to 51 °C (Δ*T* = 29 °C), but with the 60% transmittance P(EDOT/Ani) : PSS film, it only increased to 30 °C (Δ*T* = 10 °C). The temperature variation of P(EDOT/Ani) : PSS film was also smaller than that of pristine PEDOT : PSS and PAni : PSS films, whose temperature variation (ΔT) are 13 °C, 12 °C, respectively. (see Fig. 5(a)). These results effectively demonstrate that the P(EDOT/Ani) : PSS film is applicable for energy saving field. We also measured the stability of the films under harsh conditions to test the film durability. The UV light resistance of the film was measured with 254 nm wavelength light for 250 h. As shown in Fig. 6, the first column represents variation of the optical properties at 280–2700 nm wavelength (see UV-vis-NIR spectra of Fig. S3 in the ESI†). After UV irradiation for 250 h, the change in the heat shielding efficiency was 2.0% and the reduction of transmittance (at 550 nm) was 4.1%. These slight changes were caused by degradation of the polymer in UV light. The other columns show the stability test results under high temperature (85 °C) and high temperature/humidity (85 °C/85%) conditions. After 250 h, the variations in heat shielding efficiency were 2.3% and 2.6%, and the transmittance was reduced by 4.7% and 5.3% respectively. These results are encouraging because there was little change in the transmittance under the harsh conditions. Finally, a P(EDOT/Ani) : PSS heat shielding film of large surface area was fabricated by a roll-to-roll slot-die coating process. A dispersion of P(EDOT/Ani) : PSS was poured onto a PET substrate (500 mm in width × 150 m in length) and then the coating took place. Residual solvent was removed using a convection oven at 120 °C for 3 min (see Fig. 7).

## Conclusions

In conclusion, we report the successful polymerization of 3,4-ethylenedioxythiophene and aniline in aqueous medium by two-stage shot growth. A conjugated polymer complex with enhanced absorption in the near infrared area was obtained by controlling time intervals for aniline monomer addition. P(EDOT/Ani) : PSS was also successfully applied as an efficient heat shielding material. Introduction of polyaniline to PEDOT : PSS significantly improves the shielding efficiency in comparison with pristine PEDOT : PSS. The shielding efficiency of P(EDOT/Ani) : PSS film was calculated using UV-vis-NIR spectra. The total shielding efficiency increased by 11.2% at the same transmittance, which is 60% in 550 nm wavelength. We also fabricated a large-scale film (500 nm in width × 150 m in length) of P(EDOT/Ani) : PSS and conducted a heat shielding test of the film by measuring the temperature variation. Compared with the PEDOT : PSS and PAni film, the P(EDOT/Ani) : PSS film exhibited better the heat shielding effect. By employing the two-stage shot growth process, we easily synthesized the desired polymer composites. Our results indicate that P(EDOT/Ani) : PSS can facilitate commercial application of heat shielding film and simplify the preparation of nanocomposites.

## Conflicts of interest

There are no conflicts to declare.

## Supplementary Material

RA-008-C8RA01122B-s001
