# Supplementary material for: Facile synthesis of P(EDOT/Ani) : PSS with enhanced heat shielding efficiency via two-stage shot growth
Source: RSC Adv. 2018 Apr 9;8(23):12992–8. doi: 10.1039/c8ra01122b (PMC9079726; doi:10.1039/c8ra01122b)
Supplement: RA-008-C8RA01122B-s001 [file RA-008-C8RA01122B-s001.pdf]

Electronic Supplementary Material (ESI) for RSC Advances.
This journal is © The Royal Society of Chemistry 2018

Facile Synthesis of P(EDOT/Ani):PSS with Enhanced Heat Shielding Efficiency via Two-Stage Shot Growth

Chanil Park‡, Soeun Im‡, Wonseok Cho, Yunryeol Kim, and Jung Hyun Kim*

Department of Chemical and Biomolecular Engineering, Yonsei University, Yeonsero 50, Seodaemun-gu, Seoul 120–749, Republic of Korea

*E-mail: jayhkim@yonsei.ac.kr

‡These authors contributed equally to this work.

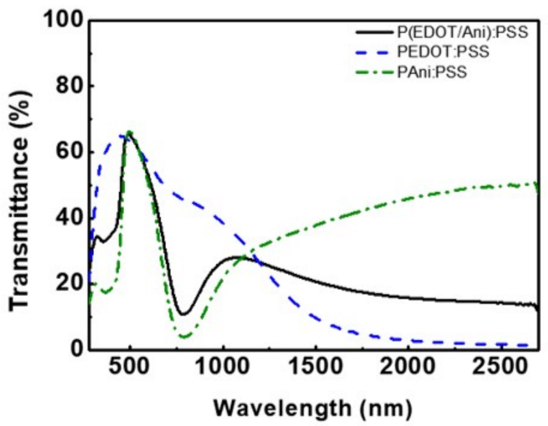

Figure S1 UV-vis-NIR transmittance of P(EDOT/Ani):PSS film, PEDOT:PSS film, and PAni:PSS film with the same transmittance, 60% at 550 nm wavelength.

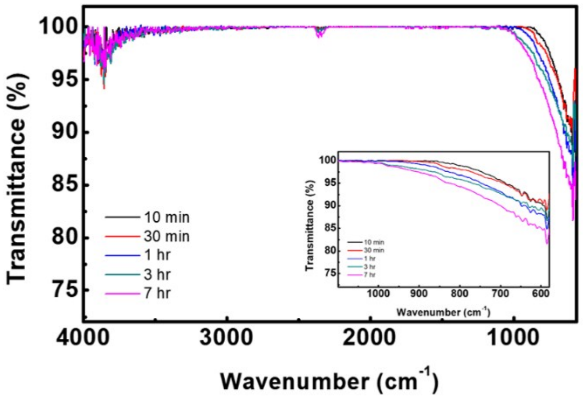

Figure S2 FT-IR spectra of P(EDOT/Ani):PSS for various time intervals of aniline monomer addition.

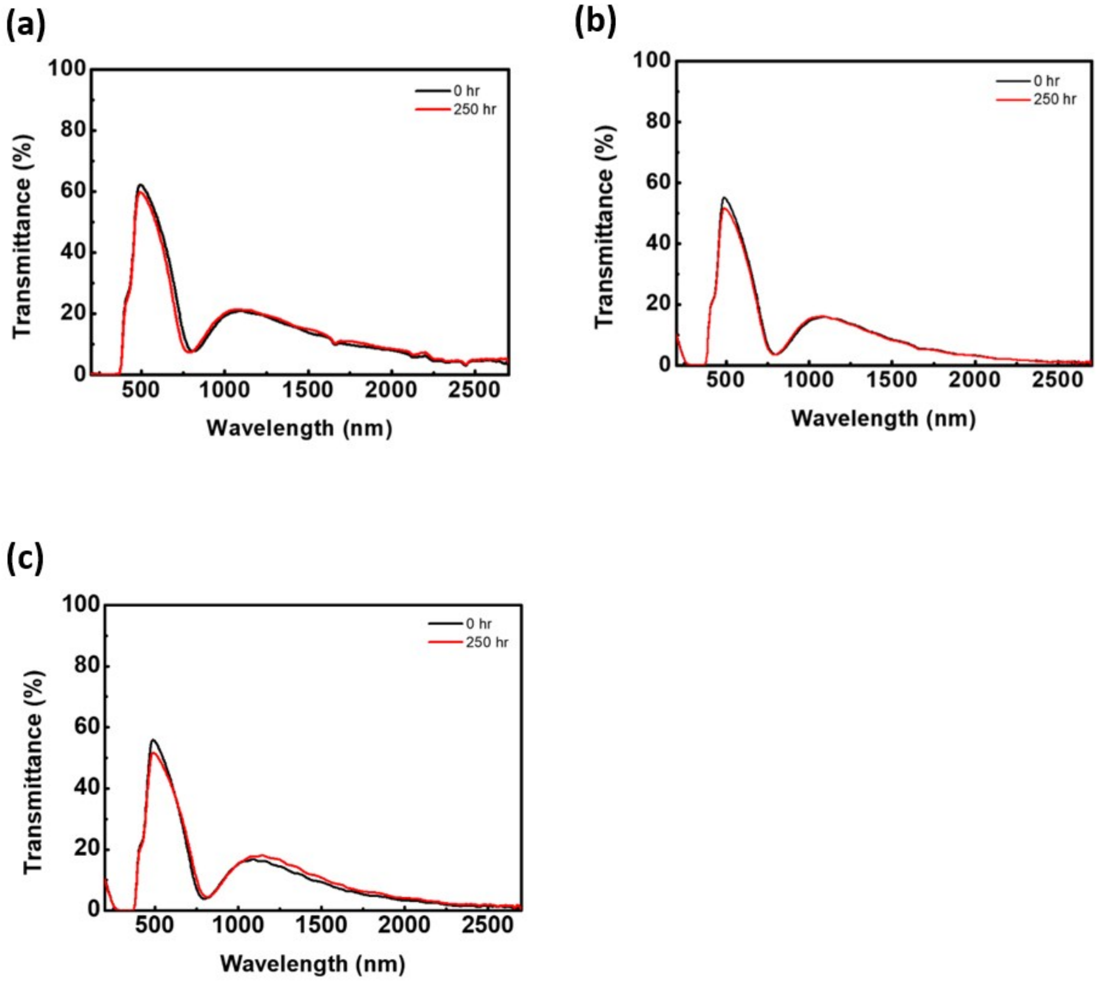

Figure S3 Transmittance spectra variation of the P(EDOT/Ani):PSS film after durability test : (a) Ultraviolet resistance test (254 nm UV radiation for 250 h), (b) high-temperature test (85℃) for 250 hours, (c) high-temperature and humidity test (85℃/ 85%) for 250 h.
